# PPP2R2A inhibition contributes to preeclampsia by regulating the proliferation, apoptosis, and angiogenesis modulation potential of mesenchymal stem cells

**DOI:** 10.1186/s13008-024-00118-w

**Published:** 2024-05-11

**Authors:** Yan Liu, Fangle Gu, Jun Gao, Yingyan Gu, Zhiyue Li, Dan Lu, Yanxin Zhang

**Affiliations:** Department of Obstetrics and Gynecology, Clinical Medical College, Yangzhou University, No. 98 Nantong West Road, Yangzhou, 225001 China

**Keywords:** PPP2R2A, Pre-eclampsia, Mesenchymal stem cells

## Abstract

**Background:**

The precise mechanisms underlying preeclampsia (PE) pathogenesis remain unclear. Mesenchymal stem cells (MSCs) are involved in the pathology of PE. The aim of our study was to identify the effects of protein phosphatase 2 regulatory subunit B α (PPP2R2A) on MSCs and ascertain its latent role in the progression of PE.

**Methods:**

Reverse-transcription quantitative polymerase chain reaction and western blot analyses were performed to determine the expression of PPP2R2A in decidual tissue and decidual (d)MSCs from healthy pregnant women and patients with PE as well as the expression levels of Bax and Bcl-2 in dMSCs. The levels of p-PI3K, PI3K, p-AKT, and AKT were determined using western blotting. Cell growth, apoptosis, and migration were analyzed using MTT, flow cytometry, and Transwell assays, respectively. Human umbilical vein endothelial cell (HUVEC) tube formation ability was assayed using a HUVEC capillary-like tube formation assay.

**Results:**

PPP2R2A was downregulated in decidual tissues and dMSCs of patients with PE when compared with that in healthy pregnant women. Moreover, upregulation of PPP2R2A enhanced cell proliferation, reduced apoptotic dMSC, inhibited Bax expression, and increased Bcl-2 levels. Conditioned medium from PPP2R2A-overexpressing dMSCs promoted HTR-8/SVneo cell migration and angiogenesis of HUVEC. Furthermore, the PPP2R2A plasmid suppressed PI3K/AKT pathway activation in dMSCs. However, these effects were partially reversed by LY2940002 treatment.

**Conclusion:**

PPP2R2A inhibition contributes to PE by regulating the proliferation, apoptosis, and angiogenesis of MSCs, providing a new therapeutic target for PE diagnosis and treatment.

**Supplementary Information:**

The online version contains supplementary material available at 10.1186/s13008-024-00118-w.

## Background

Preeclampsia (PE) is a complex pregnancy-specific disease characterized by hypertension and proteinuria during or after the second trimester of pregnancy [1, 2]. The morbidity of PE has increased to nearly 5–8%, and it is the main cause of mortality in pregnant women and newborns [3]. Numerous factors have been reported in the development and pathogenesis of PE, including excessive trophoblast apoptosis, insufficient spiral artery remodeling, endothelial dysfunction, and placental oxidative stress [4–6]. However, the precise molecular mechanisms underlying the development of PE have not yet been elucidated. Understanding the behavior of trophoblastic cells will help develop new targets for PE therapy.

Mesenchymal stem cells (MSCs) are multipotent stem cells derived from various tissues and exhibit self-renewal, expansion, and differentiation. Many studies have suggested that the therapeutic potential of MSCs is attributable to their characteristics, such as differentiation, immune regulation, and paracrine signaling cytokines [7, 8]. . MSCs are widely used to study PE in vitro. Basmaeil et al. verified that heme oxygenase-1 (HMOX1) was partly responsible for the phenotypic and functional abnormalities of MSCs from the placenta of patients with PE [9]. Gu et al. revealed that miR-30a contributes to PE by regulating the proliferation, apoptosis, and angiogenic modulation potential of MSCs by targeting apoptosis and caspase activation inhibitors (AVEN) [10]. However, little is known about the potential role of decidual (d)MSCs in the development and progression of PE.

PPP2R2A is a member of the protein phosphatase 2 regulatory subunit B family that negatively regulates cell growth and division [11]. In addition, PPP2R2A has been proven to be a tumor inhibitor involved in the proliferation, invasion, and epithelial-to-mesenchymal transition (EMT) of cancer cells, including gastric cancer [12], liver cancer [13], and ovarian cancer [14]. PPP2R2A plays a regulatory role in placental development [15]; however, the regulatory role of PPP2R2A in dMSC function remains unclear.

Thus, the aims of our study were to (i) determine the expression of PPP2R2A in the decidual tissues of patients with PE and dMSCs; (ii) explain whether PPP2R2A participates in the development of PE by regulating placental trophoblast cell proliferation, apoptosis, and angiogenesis; and (iii) analyze the molecular pathways through which PPP2R2A regulates PE progression to identify promising targets for PE therapy.

## Results

### PPP2R2A was downregulated in the decidual tissues and dMSCs of PE patients

**Fig. 1:**
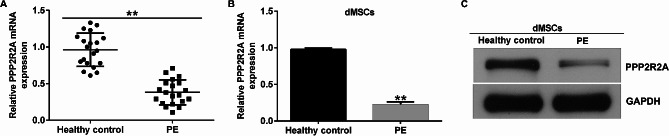
Level of PPP2R2A in the decidual tissues and dMSCs from PE patients. (**A**) RT-qPCR analysis of PPP2R2A levels in decidual tissues from patients with PE or healthy pregnant women (*n* = 20). (**B**) RT-qPCR analysis of PPP2R2A levels in dMSCs from patients with PE or healthy pregnant women (*n* = 3). (**C**) Detection of PPP2R2A expression in dMSCs from patients with PE or healthy pregnancies (*n* = 3). **indicates *P* < 0.01

We collected decidual tissues and dMSCs from 20 patients with PE and 20 healthy pregnant women. We observed a marked decrease in PPP2R2A expression in the decidual tissues of patients with PE when compared with the controls (Fig. 1A). In addition, PPP2R2A expression was remarkably reduced in the dMSCs of patients with PE at the mRNA and protein levels when compared with that in healthy pregnancies (Fig. 1B and C). Our data suggest that PPP2R2A is abnormally expressed in patients with PE and is involved in PE progression.

### PPP2R2A plasmid enhanced dMSC proliferation and reduced cell apoptosis

**Fig. 2:**
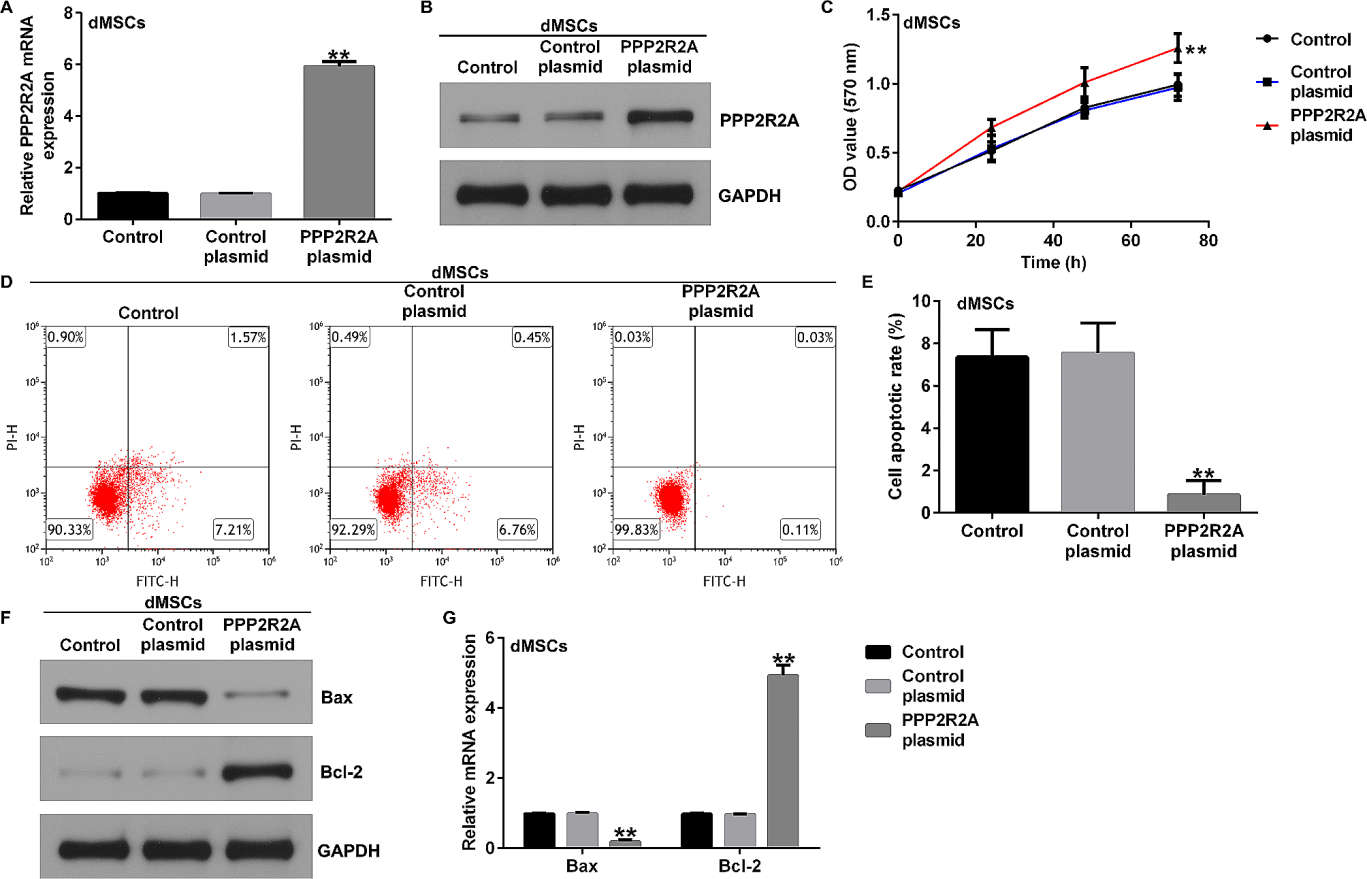
Influence of PPP2R2A plasmid on dMSC proliferation and apoptosis. The control plasmid or PPP2R2A plasmid was transfected into dMSCs for 36 h. Expression of PPP2R2A in the control plasmid and PPP2R2A plasmid groups was evaluated using (**A**) RT-qPCR analysis and (**B**) western blotting. (**C**) dMSC proliferation was assessed using the MTT assay. (**D**) Apoptotic dMSCs were determined using flow cytometry. (**E**) Quantification of dMSC apoptosis using GraphPad software. (**F**) Western blotting analysis of Bax and Bcl-2 expression levels. The mRNA levels of Bax and Bcl-2 (**G**) were analyzed using RT-qPCR. *N* = 3. ** indicates *P* < 0.01. vs. control plasmid

To further elucidate the mechanism of PPP2R2A in PE, dMSCs were transfected with the control plasmid or PPP2R2A plasmid for 48 h. As shown in Fig. 2A-B, the level of PPP2R2A was significantly higher in PPP2R2A plasmid-treated dMSCs than in the control plasmid group. Furthermore, MTT and flow cytometry assays demonstrated that upregulation of PPP2R2A enhanced the proliferation of dMSCs (Fig. 2C) and suppressed apoptosis of dMSCs (Fig. 2D-E), in comparison with the control plasmid group. We also determined the expression of apoptosis-related proteins Bax and Bcl-2 by using reverse transcription-quantitative polymerase chain reaction (RT-qPCR) and western blot assays. We found that the PPP2R2A plasmid notably suppressed Bax expression (Fig. 2F and G) and increased Bcl-2 levels (Fig. 2F and G) when compared with the control plasmid group. Our findings indicated that PPP2R2A regulates dMSC proliferation and apoptosis in PE.

### Conditioned medium from PPP2R2A-overexpressing dMSCs promoted HTR-8/SVneo cell migration

**Fig. 3:**
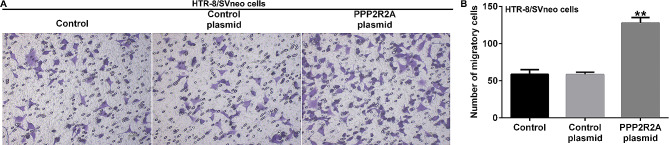
Influence of PPP2R2A-overexpressing dMSCs on HTR-8/SVneo migration. The control plasmid or PPP2R2A plasmid was transfected into dMSCs for 36 h. (**A**) Cell migration was detected using the Transwell assay. (**B**) Quantification of the migrated HTR-8/SVneo cells. *N* = 3. ** indicates *P* < 0.01 vs. control plasmid

To further illustrate the functions of PPP2R2A in HTR-8/SVneo cell migration, the control plasmid or PPP2R2A plasmid was transfected into dMSCs for 36 h. The Transwell assay revealed that HTR-8/SVneo cell migration capability was significantly enhanced in the supernatant from PPP2R2A-overexpressing dMSCs, as opposed to the control plasmid group (Fig. 3A and B). Our findings suggest that the conditioned medium from PPP2R2A-overexpressing dMSCs affected the migratory ability of HTR-8/SVneo cells.

### Conditioned medium from PPP2R2A-overexpressing dMSCs promoted angiogenesis of human umbilical vein endothelial cell (HUVEC)

**Fig. 4:**
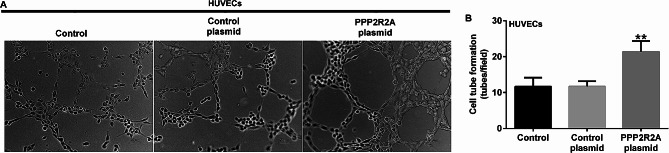
Influence of PPP2R2A-overexpressing dMSCs on HUVEC tube formation. The control plasmid or PPP2R2A plasmid was transfected into dMSCs for 36 h. (**A**) Representative images of HUVEC tube formation ability in the control plasmid or PPP2R2A plasmid group. (**B**) Detection of HUVEC tube formation ability. *N* = 3. *indicates *P* < 0.05, **indicates *P* < 0.01 vs. control plasmid

Previous studies have shown that inappropriate modification of spiral arteries may result in the development of PE [16]. Thus, we determined whether dMSCs overexpressing PPP2R2A affected angiogenesis of HUVEC by using a HUVEC capillary-like tube formation assay. As shown in Fig. 4A and B, the supernatants from PPP2R2A-overexpressing dMSCs led to more tubes. These data indicate that the conditioned medium from PPP2R2A-overexpressing dMSCs promoted angiogenesis of HUVEC.

### PPP2R2A regulated the functions of dMSCs via activating PI3K/AKT signaling pathway

**Fig. 5:**
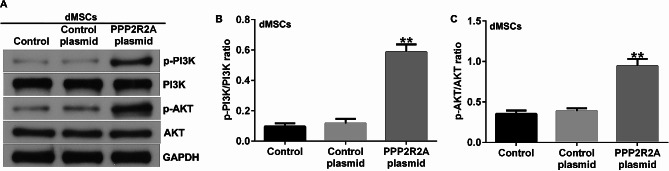
Influence of PPP2R2A plasmid on PI3K/AKT signaling pathway in dMSCs. The control plasmid or PPP2R2A plasmid was transfected into dMSCs for 48 h. (**A**) Detection of p-PI3K and p-AKT expression dMSCs transfected with the control plasmid or PPP2R2A plasmid by using western blotting. (**B**, **C**) The mRNA levels of p-PI3K and p-AKT in dMSCs treated with the control plasmid or PPP2R2A plasmid were determined using RT-qPCR. *N* = 3. **indicates *P* < 0.01 vs. control plasmid

The PI3K/AKT signaling pathway is involved in PE when aberrantly activated [17]. Western blotting was performed to determine the expression of proteins associated with the PI3K/AKT signaling pathway. We observed that the upregulation of PPP2R2A notably enhanced the protein levels of p-PI3K and p-AKT (Fig. 5A) as well as p-PI3K/PI3K and p-AKT/AKT ratios (Fig. 5B-C), in comparison with the control plasmid group. These findings indicate that PPP2R2A activates the PI3K/AKT signaling pathway in dMSCs.

### LY2940002 partly reversed the effects of PPP2R2A plasmid on PI3K/AKT signaling pathway

**Fig. 6:**
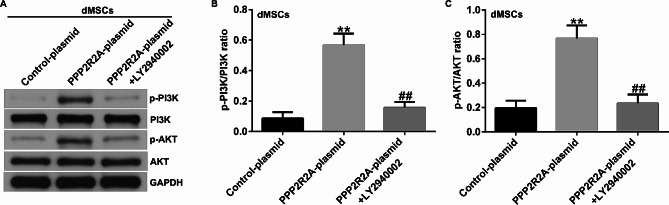
Influence of LY2940002 and PPP2R2A plasmid on PI3K/AKT signaling pathway. (**A**) Determination of p-PI3K and p-AKT. (**B**, **C**) The mRNA levels of p-PI3K and p-AKT were assessed using RT-qPCR. *N* = 3. ** indicates *P* < 0.01 vs. control plasmid; ## indicates *P* < 0.01 vs. PPP2R2A plasmid

Numerous studies have revealed the interaction between PE and the PI3K/AKT signaling pathway. In this study, dMSCs were transfected with the control plasmid or PPP2R2A plasmid and treated with a PI3K/AKT signaling pathway inhibitor (10 µM LY2940002). Western blotting results suggested that LY2940002 partially reversed the effects of the PPP2R2A plasmid on the PI3K/AKT signaling pathway in dMSCs, as confirmed by the suppression of p-PI3K and p-AKT expression (Fig. 6A) as well as reduced p-PI3K/PI3K and p-AKT/AKT ratios (Fig. 6B-C).

### LY2940002 partly reversed the effects of PPP2R2A plasmid on dMSC viability, apoptosis, HTR-8/SVneo cell migration, and HUVEC angiogenesis

**Fig. 7:**
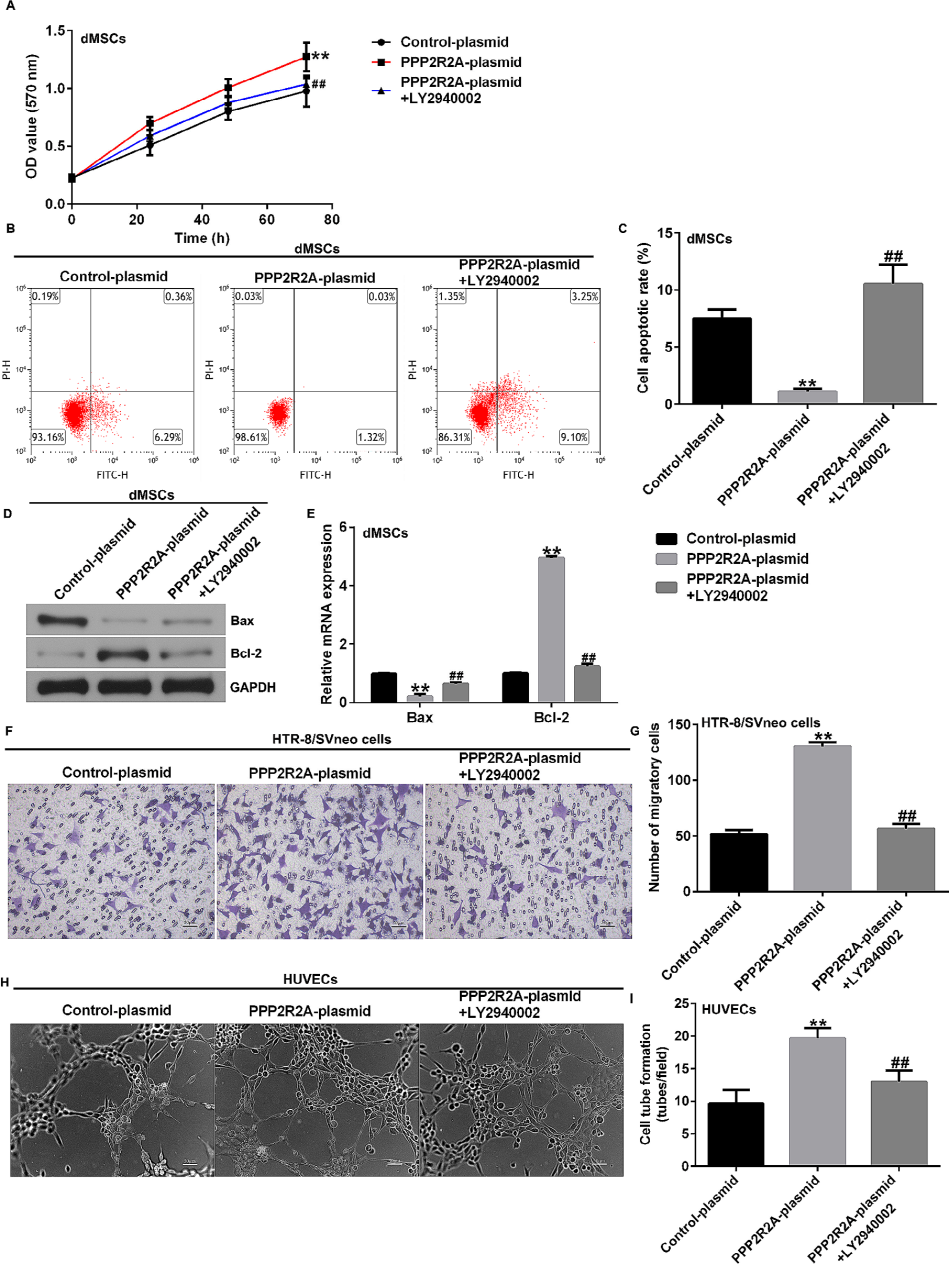
Influence of LY2940002 and PPP2R2A plasmid on dMSC proliferation, apoptosis, HTR-8/SVneo cell migration, and HUVEC tube formation. (**A**) MTT assay was performed to analyze dMSC proliferation. (**B**) dMSC apoptosis was assessed using FCM analysis. (**C**) Quantification of apoptotic dMSCs. (**D**) Western blot analysis of Bax and Bcl-2 expression. (**E**) mRNA levels of Bax and Bcl-2 were analyzed using RT-qPCR. (**F**) HTR-8/SVneo cell migration was analyzed using Transwell assay. (**G**) Migratory HTR-8/SVneo cells were calculated. (H) Tube formation of HUVEC was photographed under a microscope. (**I**) HUVEC tube formation assay was used to analyze HUVEC tube formation. *N* = 3. ** indicates *P* < 0.01 vs. control plasmid; ## indicates *P* < 0.01 vs. PPP2R2A plasmid

We investigated the effects of LY2940002 on dMSC viability, apoptosis, HTR-8/SVneo cell migration, and HUVEC angiogenesis. The results of the MTT assay and flow cytometry (FCM) analysis revealed that the upregulation of PPP2R2A enhanced cell proliferation (Fig. 7A), reduced the number of apoptotic dMSCs (Fig. 7B and C), inhibited Bax expression (Fig. 7D and E), and increased Bcl-2 levels (Fig. 7D and E). Additionally, as shown in Fig. 7F–I, improved HTR-8/SVneo migration (Fig. 7F and G) and HUVEC tube formation (Fig. 7H and I) were observed in the PPP2R2A-overexpressing dMSC culture medium treatment group. However, these effects were partially reversed by LY2940002 treatment. Thus, these findings indicate that PPP2R2A contributes to PE by regulating the proliferation, apoptosis, and angiogenesis of dMSC via the PI3K/AKT signaling pathway.

## Discussion

PE is a systemic disease unique to pregnancy and is caused by multiple factors that seriously threaten the survival of both mother and fetus. PE has many common symptoms, including an imbalance in angiogenesis and secretion of inflammatory factors [18, 19]. The decidua is a highly modified endometrium, and normal functioning is necessary for a normal pregnancy [18]. MSCs have the ability to self-renew and secrete factors that promote endogenous angiogenesis and neurogenesis and have great potential in the treatment of many diseases [20]. Recent studies have shown that placenta-derived MSCs contribute to the maturation and stability of blood vessels [21]. These findings suggest that abnormal MSCs contribute to the development of PE. However, the molecular mechanisms underlying the action of dMSC in PE remain unclear.

PPP2R2A plays a tumor-inhibitory role in various cancers. Liang et al. reported that miR-892a regulates PPP2R2A expression and promotes human colorectal cancer cell proliferation [22]. Zhu et al. have reported that lncRNA-HCG18 regulates papillary thyroid cancer cell viability, apoptosis, migration, invasion, and EMT by regulating the miR-106a-5p/PPP2R2A axis [23]. On the basis of these findings, we investigated the role of PPP2R2A in PE progression. Our findings are consistent with previous observations, indicating that PPP2R2A is downregulated in the decidual tissues and dMSC of patients with PE. Thus, we speculated that PPP2R2A in dMSC may be related to PE development and examined the regulatory effects of PPP2R2A on dMSC viability and apoptosis in PE. The control plasmid and PPP2R2A plasmid were transfected into dMSCs for 48 h, and our data suggested that the level of PPP2R2A was higher in PPP2R2A plasmid-treated dMSCs than in the control plasmid group. Moreover, upregulation of PPP2R2A enhanced the proliferation of dMSCs and suppressed apoptosis, which may trigger the onset of PE. Bax is a pro-apoptotic member of the Bcl-2 family that induces apoptosis when overexpressed [24]. Previous studies have shown a significant increase in Bax expression in patients with PE [25]. Naseri et al. revealed the roles of Bax and Bcl-2 in 3-NC mediated apoptosis of human cancer cells [26]. We examined Bax and Bcl-2 in PPP2R2A-overexpressing dMSCs. RT-qPCR and western blot analyses showed that the PPP2R2A plasmid reduced Bax expression at both mRNA and protein levels in dMSCs, whereas it significantly increased the level of the anti-apoptotic Bcl-2 protein. Collectively, these data suggested that PPP2R2A-overexpressing dMSCs affected proliferation and apoptosis of dMSCs by regulating PPP2R2A expression.

Severe PE is closely related to a decline in trophoblast invasion and uterine spiral arteriole recasting disorders [27]. Cheng et al. confirmed the role of lncRNA Linc00261 in PE and its effect on trophoblast invasion and migration via regulation of the miR-558/TIMP4 signaling pathway [28]. Chi et al. reported that LINC00473 downregulation facilitates trophoblast migration and invasion via the miR-15a-5p/LITAF axis in PE [29]. Therefore, we investigated whether dMSCs expressing PPP2R2A affect the migration ability of HTR-8/SVneo cells and the formation of capillaries and networks. In this study, we found that dMSC culture supernatants overexpressing PPP2R2A enhanced the migration of HTR-8/SVneo cells and angiogenic ability of HUVEC. In this study, we found that PPP2R2A overexpression stimulated the migration of HTR-8/SVneo cells and promoted HUVEC tube formation, suggesting that PPP2R2A is involved in PE progression through regulation of dMSC proliferation, apoptosis, migration, and angiogenesis.

PPP2R2A has been suggested to be involved in cellular biological processes through many signaling pathways, such as the AKT/mTOR and ERK1/2 pathways [30, 31]. Moreover, the PI3K/AKT signaling pathway is involved in PE progression [17, 32]. Wang et al. suggested that the transfer of miR-15a-5p by placental exosomes promotes PE progression by regulating the PI3K/AKT signaling pathway via CDK1 [17]. Han et al. confirmed that miR-483 was downregulated in PE by targeting IGF1 and regulating the PI3K/Akt/mTOR pathway in endothelial progenitor cells [32]. However, the molecular mechanisms underlying human trophoblast invasion remain largely unknown. Therefore, we investigated whether the AKT signaling pathway is involved in the cell invasion behavior of PE by measuring the levels of p-PI3K and p-AKT and treating dMSCs with the PPP2R2A plasmid by using the PI3K/AKT signaling pathway inhibitor LY2940002. Our results confirmed that LY2940002 partially reversed the effect of the PPP2R2A plasmid on dMSC proliferation, apoptosis, migration, and regulation of angiogenesis, as confirmed by reduced p-PI3K and p-AKT levels as well as the inhibition of cell proliferation, promotion of apoptotic dMSC cells, suppression of HTR-8/SVneo migration, and HUVEC tube formation.

In summary, our findings are the first to reveal a potential role of PPP2R2A in the pathogenesis of PE. Mechanistic studies have indicated that PPP2R2A inhibition contributes to PE by regulating the proliferation, apoptosis, and angiogenesis of dMSC. However, this study has some limitations. Although some interesting phenomena have been associated with PPP2R2A, it remains unclear whether PPP2R2A inhibition can reverse the development of PE symptoms in vivo. We were unable to cover all other signaling pathways involved in PPP2R2A. Our findings suggest that PPP2R2A could serve as a potential therapeutic marker for PE.

## Materials and methods

### Clinical samples

Human decidual tissues from patients with PE (*n* = 20) and healthy pregnant women (*n* = 20) were aseptically obtained during cesarean section at the Clinical Medical College, Yangzhou University. The characteristics of the patients with preeclampsia are listed in Table 1. This study was approved by the Ethics Committee of the Clinical Medical College, Yangzhou University, in accordance with the Declaration of Helsinki. Written informed consent was obtained from the patients prior to surgery. PE was defined as the presence of hypertension and proteinuria after 20 weeks of gestation. Elevated blood pressure (systolic pressure > 140 mmHg or diastolic pressure > 90 mmHg) was considered as hypertension. The exclusion criteria included multiple pregnancies, pregnancy diabetes, chronic nephritis, liver disease, chronic hypertension, HELLP syndrome, and other infectious or tumorous diseases.

**Table 1:** Characteristics of patients with preeclampsia and healthy controls

Parameters	PE (n = 20)	Healthy control (n = 20)
Maternal age (years)	26–34	25–33
Gestational weeks at delivery	34–37	38–41
Systolic Blood Pressure (mmHg)	160.4 ± 7.2	115.2 ± 5.1
Diastolic Blood Pressure (mmHg)	101.5 ± 5.3	73.5 ± 6.2
Proteinuria (g/24 h)	2.4 ± 0.5	Negative

### dMSC isolation and culture

dMSCs were isolated from the decidual tissues of the placentas of patients with PE and healthy pregnancies. The decidual tissues were washed twice with phosphate-buffered saline (PBS; 70,013,032; Gibco, Grand Island, NY, USA). The decidual tissue was then mechanically broken, incubated in the enzyme mixture (5 U/mL hyaluronidase, 125 U/mL collagenase, and 50 U/mL dispase; Sigma, St. Louis, MO, USA), and stirred gently at 37 °C for 1 h. Then, they were suspended in DMEM/F12 culture solution containing 10% FBS in an incubator containing 5% CO_2_ at 37 °C. When the cells reached approximately 70% confluency, they were separated using 0.25% trypsin and transferred to new culture plates. FCM was used to detect specific phenotypic surface antigens on MSCs.

### Cell culture

HTR-8/SVneo and HUVEC cells were acquired from the ATCC and maintained in RPMI-1640 medium supplemented with 10% FBS (30,067,334; Thermo Fisher Scientific, Waltham, MA, USA) and 1% penicillin/streptomycin (15,140,122; Gibco, Grand Island, NY, USA) in an incubator containing 5% CO_2_ at 37 °C.

### Cell transfection

Overexpression of PPP2R2A in dMSCs was achieved by transfecting cells with the PPP2R2A plasmid and control plasmid by using Lipofectamine® 3000 reagent (L3000015; Thermo Fisher Scientific, Waltham, MA, USA) for 48 h at 37 ℃, according to the protocol. Cell transfection efficiency was assessed using RT-qPCR, and the supernatants were obtained for subsequent experiments.

### RT-qPCR analysis

The levels of PPP2R2A, Bax, and Bcl-2 were measured using RT-qPCR. Total RNA from dMSCs was extracted using TRIzol reagent (15596026CN; Invitrogen, Carlsbad, CA, USA), and the total RNA was treated with DNase I to digest the genomic DNA. The cDNA was synthesized using a Reverse Transcription Kit (A54985; Invitrogen), followed by PCR amplification using an ABI PRISM 7900 sequence detection system (Applied Biosystems, USA). Target gene expressions were obtained using 2^−ΔΔCt^ method. The primer sequences used for PCR are listed in Table 2.

**Table 2:** Primer sequences for PCR

Gene	Primer sequences
PPP2R2A Bcl-2 Bax GAPDH	forward, 5′-GCAACAGGAGATAAAGGTGGTAG-3′; reverse, 5′-TGGTTCATGGCTCTGGAAGGTG-3′; forward, 5′-AGGATTGTGGCCTTCTTTGAG-3′; reverse, 5′-AGCCAGGAGAAATCAAACAGAG-3′; forward, 5′-TCTGAGCAGATCATGAAGACAGG-3′; reverse, 5′-ATCCTCTGCAGCTCCATGTTAC-3′; forward, 5′-TTTGGTATCGTGGAAGGACTC-3′; reverse, 5′-GTAGAGGCAGGGATGATGTTCT-3′.

### Western blot assay

The dMSCs were lysed using RIPA buffer (AS1004; ASPEN) for 30 min on ice. Proteins were resolved using an SDS-PAGE kit (AS1012; ASPEN) and transferred onto PVDF membranes. Then, the membranes were blocked in 5% skim milk for 2 h at room temperature and incubated in specific antibodies against GAPDH (ab181602; 1:10000 dilution; Abcam, Cambridge, MA, USA), PPP2R2A (16569-1-AP; 1:1000 dilution; Wuhan Sanying Biotechnology, Wuhan, China), Bax (60178-1-Ig; 1:1000 dilution; Wuhan Sanying Biotechnology), Bcl-2 (50599-2-Ig; 1:1000 dilution; Wuhan Sanying Biotechnology), p-PI3K (ab182651; 1:500 dilution; Abcam), PI3K (ab191606; 1:1000 dilution; Abcam), p-AKT (#4060; 1:1000 dilution; CST, Danvers, MA, USA), or AKT (#4691; 1:3000 dilution; CST) overnight at 4 ℃. Then, the membranes were incubated with secondary antibodies for 1 h. Finally, signals were developed using ECL detection reagents (Pierce Biotechnology), according to the manufacturer’s instructions.

### MTT assay

The dMSCs were cultured in 96-well plates at 37 ℃. The MTT solution was added to each well and incubated for 4 h. Then, the solution was dislodged, and 100 µL DMSO was added to each well to solubilize the formazan product. The OD_570_ value of each well was measured using a microplate reader (BioTek, USA) after 15 min of vibration mixing, according to the manufacturer’s instructions.

### FCM analysis

After transfection for 48 h, apoptotic dMSCs were trypsinized and stained using an Annexin V-fluorescein isothiocyanate/propidium iodide apoptosis detection kit (C1062M; Beyotime, Shanghai, China), according to the manufacturer’s instructions. A flow cytometer (BD Biosciences, USA) was used to calculate cell apoptosis, and the results were analyzed using CellQuest software.

### Cell migration assay

After transfection for 36 h, dMSCs were acquired and cultured in the bottom chamber of Transwell chambers without Matrigel, whereas HTR-8/SVneo cells were cultured in 24-well plates containing 500 µL of RPMI-1640 medium and 10% FBS (upper chamber). After cultivation for 48 h, HTR-8/SVneo cells remaining in the upper chamber (non-migrated cells) were removed using a cotton swab. Cells that adhered to the lower surface of the membrane were fixed with 4% paraformaldehyde and stained with 0.1% crystal violet at room temperature for 10 min. Migratory cells in five random fields were counted using an inverted microscope (Nikon).

### HUVEC tube formation assay

Capillary tube formation by HUVECs was measured using Matrigel. After transfection for 36 h, the dMSC culture supernatant was collected from each group. After Matrigel solidification, HUVEC and the same volume of cell supernatant were inoculated onto the Matrigel and incubated for 8 h. Image Pro Plus software was used to calculate five randomly selected fields.

### Statistical analysis

All experiments were performed at least three times. Statistical analyses were conducted using GraphPad Prism software (version 6.0). All findings are displayed as mean ± standard deviation (SD). Mean differences among groups were estimated using an unpaired two-tailed Student’s *t*-test or one-way ANOVA. Statistical significance was set at *P* < 0.05.

## Electronic supplementary material

Below is the link to the electronic supplementary material.

Supplementary Material 1

## Data Availability

The datasets used and/or analyzed during the current study are available from the corresponding author on reasonable request.
