# Correction: PPP2R2A inhibition contributes to preeclampsia by regulating the proliferation, apoptosis, and angiogenesis modulation potential of mesenchymal stem cells

**DOI:** 10.1186/s13008-025-00154-0

**Published:** 2025-06-16

**Authors:** Yan Liu, Fangle Gu, Jun Gao, Yingyan Gu, Zhiyue Li, Dan Lu, Yanxin Zhang

**Affiliations:** Department of Obstetrics and Gynecology, Clinical Medical College, Yangzhou University, No. 98 Nantong West Road, Yangzhou, 225001 China

**Correction to: Cell Division (2024) 19:18**

10.1186/s13008-024-00118-w

In this article [1], the FCM analysis representative image (Fig. 2D-control-plasmid group; Fig. 7B-PPP2R2A-plasmid + LY2940002 group) was incorrectly used by confusion. To ensure the rigor of the data, the alternative images from other independently replicated experiments are provided and modified the Figures accordingly. The Figs. 2D and 7B have been changed in this correction, and these changes do not affect the results and conclusions of the article.

Incorrect Fig. 2.

**Fig. 2:**
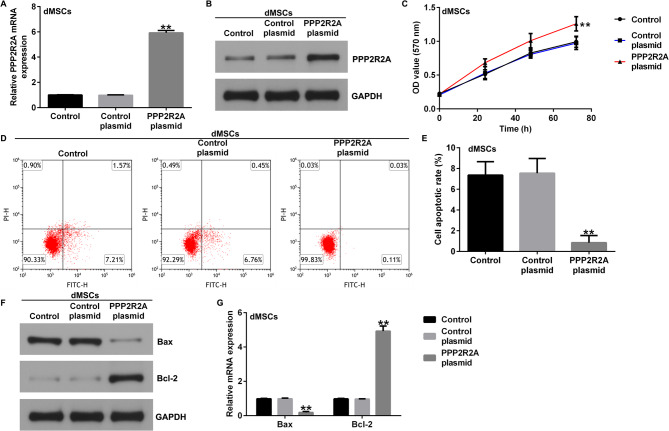
Influence of PPP2R2A plasmid on dMSC proliferation and apoptosis. The control plasmid or PPP2R2A plasmid was transfected into dMSCs for 36 h.Expression of PPP2R2A in the control plasmid and PPP2R2A plasmid groups was evaluated using (**A**) RT-qPCR analysis and (**B**) western blotting. **C** dMSCproliferation was assessed using the MTT assay. **D** Apoptotic dMSCs were determined using flow cytometry. **E** Quantification of dMSC apoptosis usingGraphPad software. **F** Western blotting analysis of Bax and Bcl-2 expression levels. The mRNA levels of Bax and Bcl-2 **G** were analyzed using RT-qPCR.N = 3. ** indicates P < 0.01. vs. control plasmid

Correct Fig. 2.

**Fig. 2:**
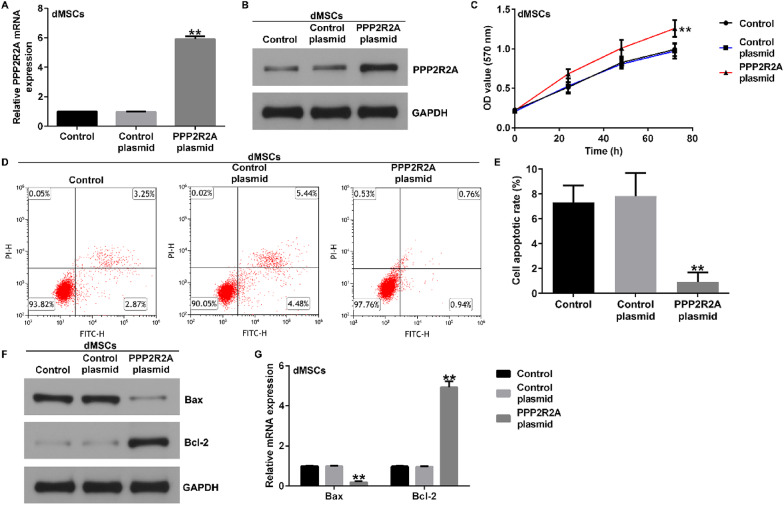
Influence of PPP2R2A plasmid on dMSC proliferation and apoptosis. The control plasmid or PPP2R2A plasmid was transfected into dMSCs for 36 h.Expression of PPP2R2A in the control plasmid and PPP2R2A plasmid groups was evaluated using (**A**) RT-qPCR analysis and (**B**) western blotting. **C** dMSCproliferation was assessed using the MTT assay. **D** Apoptotic dMSCs were determined using flow cytometry. **E** Quantification of dMSC apoptosis usingGraphPad software. **F** Western blotting analysis of Bax and Bcl-2 expression levels. The mRNA levels of Bax and Bcl-2 **G** were analyzed using RT-qPCR.N = 3. ** indicates P < 0.01. vs. control plasmid

Incorrect Fig. 7.

**Fig. 7:**
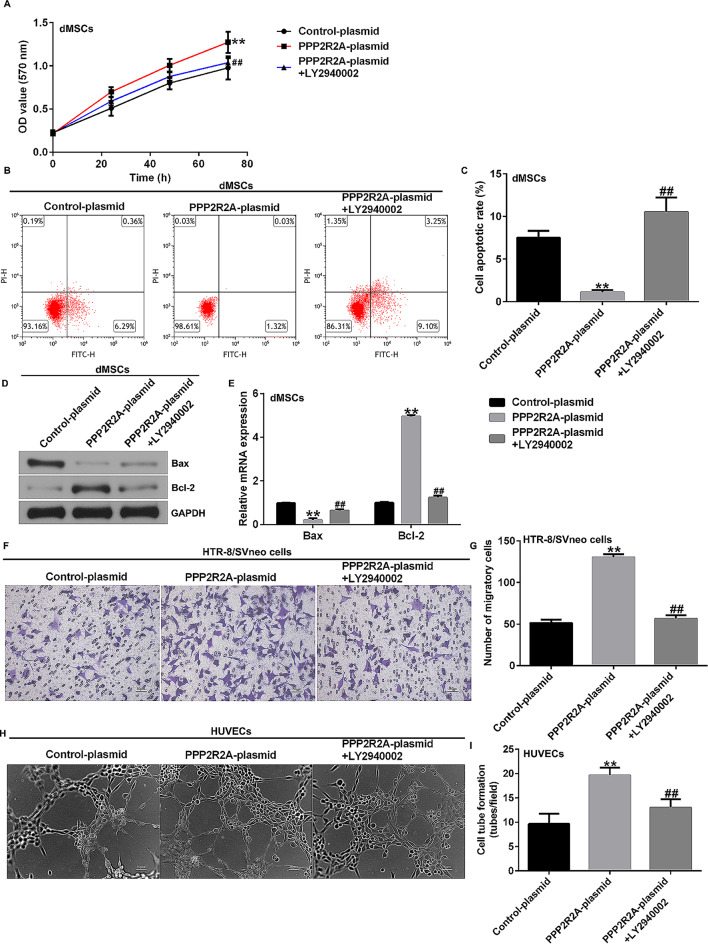
Influence of LY2940002 and PPP2R2A plasmid on dMSC proliferation, apoptosis, HTR-8/SVneo cell migration, and HUVEC tube formation. **A** MTTassay was performed to analyze dMSC proliferation. **B** dMSC apoptosis was assessed using FCM analysis. **C** Quantification of apoptotic dMSCs. **D** Westernblot analysis of Bax and Bcl-2 expression. **E** mRNA levels of Bax and Bcl-2 were analyzed using RT-qPCR. **F** HTR-8/SVneo cell migration was analyzedusing Transwell assay. **G** Migratory HTR-8/SVneo cells were calculated. **H** Tube formation of HUVEC was photographed under a microscope. **I** HUVECtube formation assay was used to analyze HUVEC tube formation. N = 3. ** indicates P < 0.01 vs. control plasmid; ## indicates P < 0.01 vs. PPP2R2A plasmid

Correct Fig. 7.

**Fig. 7:**
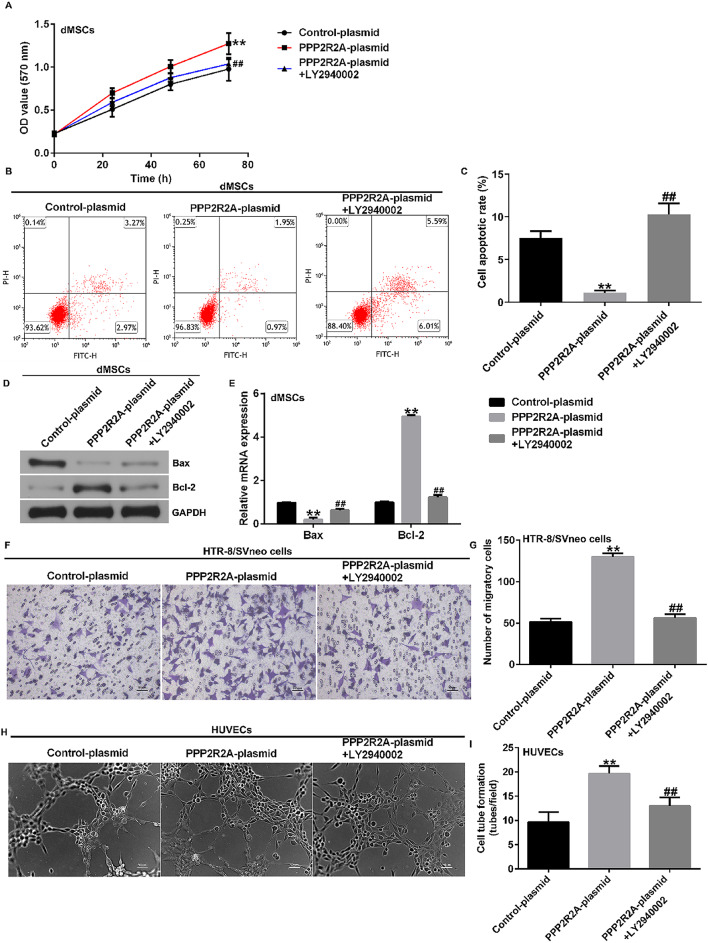
Influence of LY2940002 and PPP2R2A plasmid on dMSC proliferation, apoptosis, HTR-8/SVneo cell migration, and HUVEC tube formation. **A** MTTassay was performed to analyze dMSC proliferation. **B** dMSC apoptosis was assessed using FCM analysis. **C** Quantification of apoptotic dMSCs. **D** Westernblot analysis of Bax and Bcl-2 expression. **E** mRNA levels of Bax and Bcl-2 were analyzed using RT-qPCR. **F** HTR-8/SVneo cell migration was analyzedusing Transwell assay. **G** Migratory HTR-8/SVneo cells were calculated. **H** Tube formation of HUVEC was photographed under a microscope. **I** HUVECtube formation assay was used to analyze HUVEC tube formation. N = 3. ** indicates P < 0.01 vs. control plasmid; ## indicates P < 0.01 vs. PPP2R2A plasmid
