# Supplementary material for: PPP2R2A inhibition contributes to preeclampsia by regulating the proliferation, apoptosis, and angiogenesis modulation potential of mesenchymal stem cells
Source: Cell Div. 2024 May 11;19:18. doi: 10.1186/s13008-024-00118-w (PMC11088123; doi:10.1186/s13008-024-00118-w)
Supplement: Supplementary file 1 — Supplementary Material 1 [file 13008_2024_118_MOESM1_ESM.docx]

**Original blots of Western blot assay:**

Note:

During the western blot assay, we first cut out the corresponding membrane according to the molecular weight of the target protein and then incubate with the primary antibody. Therefore, the original imprint is not a full film. We guarantee that we strictly followed the experimental operating procedures of western blot assay.

**Figure 1**

**H: Healthy control; P: PE**


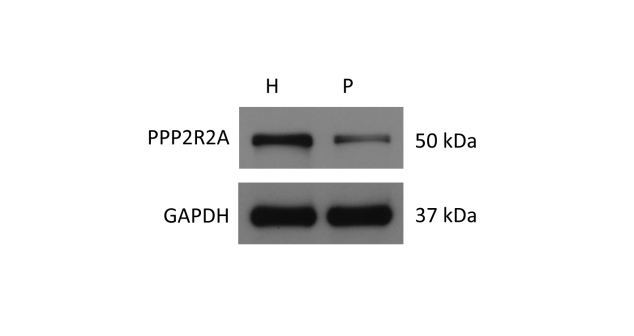


**Figure 2B**

**A: Control; B: Control-plasmid; C: PPP2R2A-plasmid**


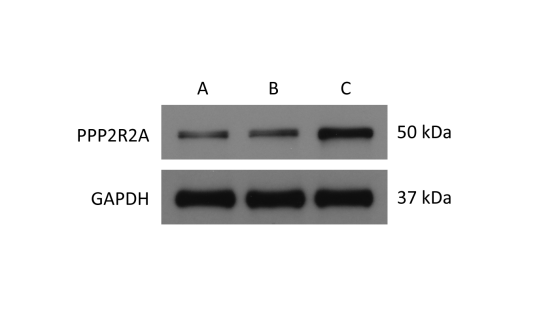


**Figure 2F**

**A: Control; B: Control-plasmid; C: PPP2R2A-plasmid**


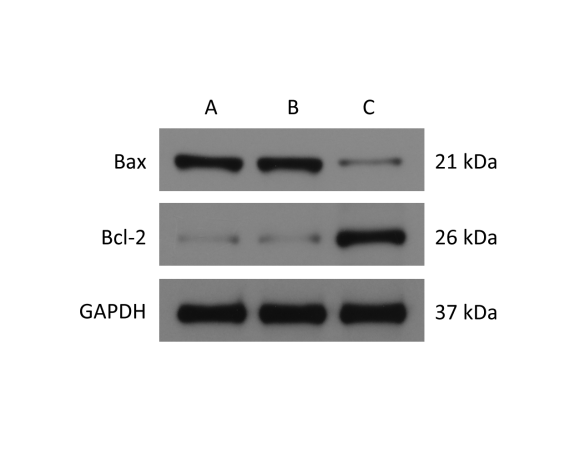


**Figure 5**

**A: Control; B: Control-plasmid; C: PPP2R2A-plasmid**

**
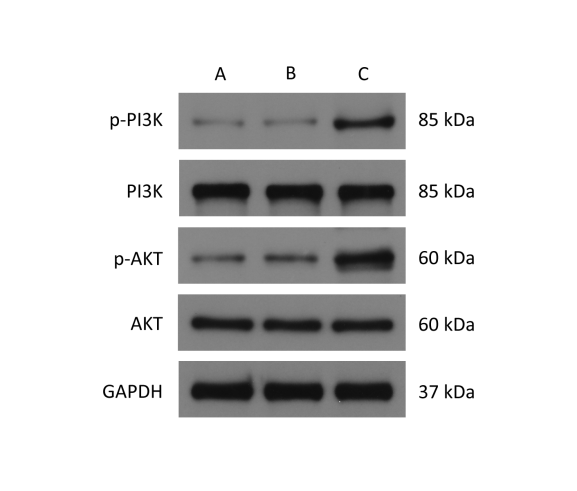
**

**Figure 6**

**B: Control-plasmid; C: PPP2R2A-plasmid; D: PPP2R2A-plasmid+LY2940002**

**
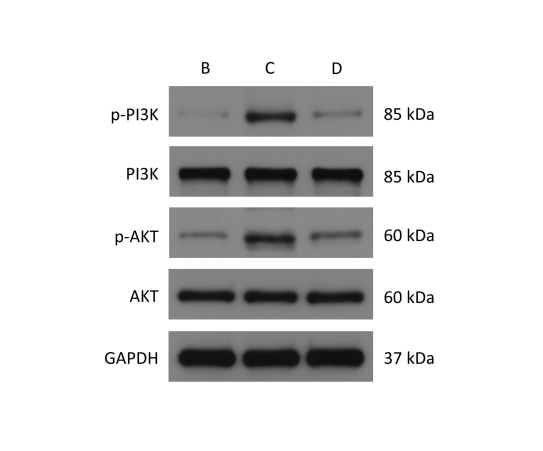
**

**Figure 7**

**B: Control-plasmid; C: PPP2R2A-plasmid; D: PPP2R2A-plasmid+LY2940002**

**
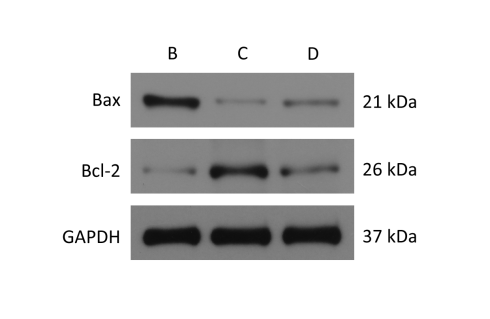
**
